# Characterization of the Composition and Biological Activity of the Venom from *Vespa bicolor* Fabricius, a Wasp from South China

**DOI:** 10.3390/toxins14010059

**Published:** 2022-01-14

**Authors:** Yong-Hua Wu, Yu Zhang, Dan-Qiao Fang, Jing Chen, Jing-An Wang, Lin Jiang, Zhu-Fen Lv

**Affiliations:** 1 Guangdong Provincial Key Laboratory of Advanced Drug Delivery Systems, Guangdong Pharmaceutical University, Guangzhou 510006, China; wuyonghuaJF@163.com (Y.-H.W.); kanola2022@163.com (D.-Q.F.); 2 Guangdong Technology Research Center for Advanced Chinese Medicine, Sun Yat-Sen University, Guangzhou 510006, China; zhangy999@mail2.sysu.edu.cn (Y.Z.); chenj633@mail2.sysu.edu.cn (J.C.); 3 Production and Research Base for Wasp Deinsectization, Guangdong Huxin Biotech Technology Co., Ltd., Jiangmen 529245, China; wangjingan@hufenghuang.com

**Keywords:** chromatography, gel electrophoresis, in vitro effect, mass, major component, wasp venom

## Abstract

We analyzed, for the first time, the major components and biological properties of the venom of *Vespa bicolor*, a wasp from South China. Using HPLC and SDS-PAGE, combined with LC–MS/MS, MALDI-TOF-MS, and NMR data to analyze *V. bicolor* venom (VBV), we found that VBV contains three proteins (hyaluronidase A, phospholipase A1 (two isoforms), and antigen 5 protein) with allergenic activity, two unreported proteins (proteins 5 and 6), and two active substances with large quantities (mastoparan-like peptide 12a (Vb-MLP 12a), and 5-hydroxytryptamine (5-HT)). In addition, the antimicrobial activity of VBV was determined, and results showed that it had a significant effect against anaerobic bacteria. The minimum inhibitory concentration and minimum bactericidal concentration for *Propionibacterium acnes* were 12.5 µg/mL. Unsurprisingly, VBV had strong antioxidant activity because of the abundance of 5-HT. Contrary to other *Vespa* venom, VBV showed significant anti-inflammatory activity, even at low concentrations (1 µg/mL), and we found that Vb-MLP 12a showed pro-inflammatory activity by promoting the proliferation of RAW 264.7 cells. Cytotoxicity studies showed that VBV had similar antiproliferative effects against all tested tumor cell lines (HepG2, Hela, MCF-7, A549, and SASJ-1), with HepG2 being the most susceptible. Overall, this study on VBV has high clinical importance and promotes the development of *Vespa bicolor* resources.

## 1. Introduction

The venom of social wasps is secreted by the venom gland of the female wasp as an important tool for hunting prey, defense, and repelling predators from their nests [1,2]. Wasp venom has attracted considerable interest due to its antimicrobial, antihistamine, anti-inflammatory, and antioxidant activities [2,3,4,5,6,7]. It has been used as a therapeutic tool in oriental medicine to treat several diseases such as wasp sting allergies, rheumatoid arthritis, and cardiovascular system diseases. The bioactive compounds of wasp venom can be divided into (i) proteins such as hyaluronidase, phospholipase, and antigen 5 protein; (ii) peptides such as mastoparan; and (iii) biologically active amines such as 5-hydroxytryptamine (5-HT) [8]. Most components of *Vespa* venom exhibit complex pharmacological activities. First, hyaluronidase, phospholipase, and antigen 5 are the three main allergenic proteins in *Vespa* venom [9,10,11,12]. They can induce IgE-mediated allergic reactions with varying degrees of cross-reaction [13] In particular, antigen 5 has been identified as the greatest allergen protein in the Vespidae family [9,14]. Nevertheless, hyaluronidase, also known as the “spreading factor,” can hydrolyze the intercellular matrix (hyaluronic acid) to facilitate venom distribution [11]. Rungsa et al. have found that *V. tropica* contains 2.5 times more hyaluronidase than *V. affinis*, making *V. tropica* venom more potent [15]. In addition, although phospholipase can cause severe hemolysis and local inflammation by disrupting the biofilm structure [7,16], it can activate smooth muscle contraction, platelet aggregation, and cell proliferation in vivo [15,16,17]. Antigen 5 protein is also involved in physiological processes such as reproduction and cancer [14,18]. Second, mastoparan is the most abundant peptide family in *Vespa* venom [19]. Several studies also have reported that mastoparan has a complex spectrum of biological effects, such as antibacterial, anticancer, and potassium-channel-blocking activities, and releases histamine as a pro-inflammatory mediator at a low concentration [20,21,22,23,24]. Thus, mastoparan may be one of the most important active components and the key component of *Vespa* venom induced inflammation. Furthermore, it exhibits differences in different *Vespa* species. For example, Lin et al. found that mastoparan of six *Vespa* species have exponential differences in mast cell degranulation activity [25]. Jalaei et al. found that mastoparan from *V. orientalis* did not cause histamine release compared with that of other species [4]. Third, 5-HT, an amine widely distributed in arthropod venom, is a recognized neurotransmitter in the human body that can affect cognitive function and emotional processing. A recent study found that the 5-HT from *V. velutina nigrithorax* has a potent activity to scavenge free radicals [5]. Overall, *Vespa* venom contains various components that have complex pathophysiological effects, and the toxicities/activities of the venom are specifically due to composition differences between species [20,26]. Identifying the components and biological activities of the venom of wasp species has high clinical importance.

*Vespa bicolor* Fabricius is a kind of social wasp present in most provinces of China [27]. Several features make it interesting to researchers. First, in recent years, the widely cultivated of *V. bicolor* for biological control result in the resources of *V. bicolor* have grown enormously in China. Second, *V. bicolor* has begun to be applied as apitherapy and medicinal liquor for rheumatism [28]. Hence, the administration of V. bicolor venom (VBV) may generally have a potential medicinal value. However, scientific theoretical basis for its clinical application is still lacking. VBV presents two peptides with antimicrobial activity (MP-VBs and VESP-VBs), a serine protease inhibitor (bicolin), and a peptide with smooth muscle contraction activity (vespin-BF) [27,29,30]. However, the compositions and biological properties of VBV have never been reported in detail to date. The allergenic/active components and biological activity of VBV remain to be elucidated, which inevitably limits the wide application of *V. bicolor*, resulting in a waste of resources.

The purpose of this study was to investigate the compositions and biological properties of VBV in detail. HPLC and SDS-PAGE combined with LC–MS/MS, MALDI-TOF-MS, and NMR data were used to identify and characterize the components of VBV. Moreover, the antimicrobial, antioxidant, anti-inflammatory, and cytotoxic activities of VBV were tested. Our work provides theoretical data support for the further application and development of VBV.

## 2. Results

### 2.1. Protein Characterization of VBV

**Figure 1:**
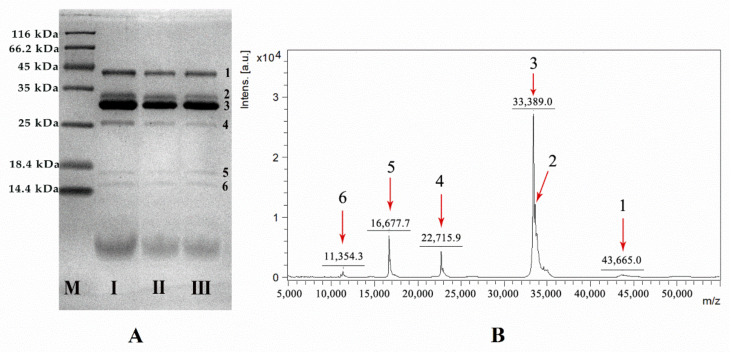
Molecular weight analysis of proteins from *Vespa bicolor* venom (VBV) was carried out by SDS-PAGE (**A**) and MALDI-TOF MS (**B**). I, II, III, and M (**A**) represent the three batches of independent VBV samples from three places of origin and marker, respectively. Numbers 1–6 represent the six proteins in VBV solution, respectively. The results of SDS-PAGE were matched with those of MALDI-TOF MS analysis.

Six proteins (larger than 10 kDa) were found in VBV at SDS-PAGE analysis, named protein 1, protein 2, protein 3, protein 4, protein 5, and protein 6, respectively (Figure 1A). Additionally, the results of MALDI-TOF-MS (Figure 1B) were consistent with those of SDS-PAGE. Six peaks appeared in the range of 10–50 kDa, suggesting that the molecular weights of proteins 1–6 in VBV were 43,665 Da, 33 kDa, 33,389 Da, 22,715 Da, 16,677 Da, and 11,354 Da, respectively.

After a data search using Mascot, proteins 1 and 4 were identified as hyaluronidase A (Supplementary Materials Figure S1A) and allergen 5, respectively (Supplementary Materials Figure S1B). Of note, proteins 2 and 3 were identified as phospholipase A1 (Supplementary Materials Figure S1C,D). Additionally, no protein valid matched with proteins 5 and 6 in the NCBI database (Supplementary Materials Figure S2), indicating that proteins 5 and 6 were newly discovered proteins.

### 2.2. Chemical Characterization of VBV

**Figure 2:**
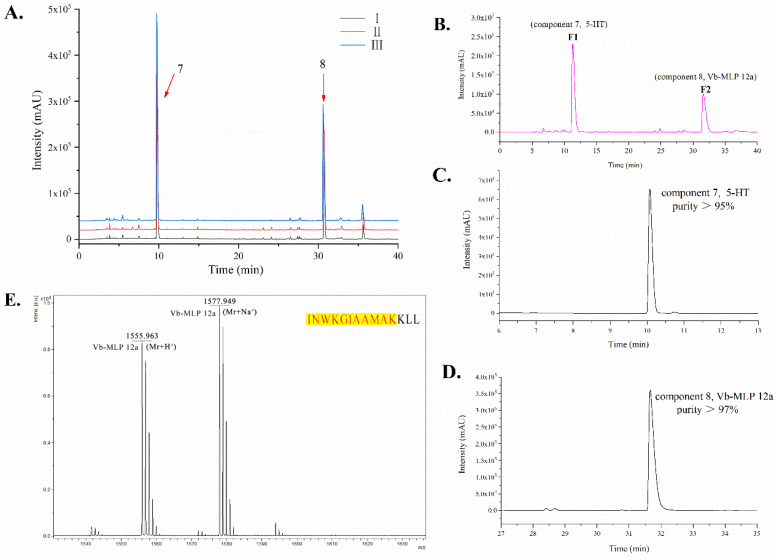
Chemical characterization of VBV. (**A**) Three batches of independent VBV samples from three places of origin were analyzed by HPLC. I, II, and III represent VBV from Guangdong, Guangxi, and Guizhou provinces, respectively. Numbers 7 and 8 represent two main components in the VBV solution. (**B**) Two main fractions (F1, F2) were separated by semi-preparative HPLC. (**C**) F1 matched to component 7 by HPLC analysis, and purity of greater than 95% was calculated by the area normalization method. (**D**) F2 matched to component 8 by HPLC analysis, and purity of greater than 97% was calculated by the area normalization method. The detection wavelength of Figure A to D was 276 nm. (**E**) The molecular weight of component 8 was analyzed by MALDI-TOF-MS, identified as mastoparan-like peptide 12a (Vb-MLP 12a) combined with LC–MS/MS results. Amino acid sequences of Vb-MLP 12a (INWKGIAAMAKKLL) are shown (upper right), sequence coverage of component 8 compared with Vb-MLP 12a as shown in the highlight.

On the basis of the HPLC pattern, two main peaks were separated from three places of origin of VBV, named compound 7 and compound 8 (Figure 2A). To separate and purify the different components of VBV, we used the semi-preparative HPLC technique. Two main fractions were obtained, and the results showed that two fractions matched components 7 and 8 in HPLC (Figure 2B). Furthermore, the purity of components 7 and 8 was greater than 95% and 97%, respectively, as calculated by the area normalization method (Figure 2C,D).

Compound 8 was analyzed by MALDI-TOF-MS. The ion at *m*/*z* 1555.963 was the quasi-molecular ion (Mr + H^+^) of compound 8 (Figure 2E), which was a peptide. Combined with the LC–MS/MS identification results of VBV, component 8 was found to be mastoparan-like peptide 12a (Vb-MLP 12a) [31] (Supplementary Material Figure S4), and its amino acid sequence is INWKGIAAMAKKLL.

In addition, compound 7 was analyzed as 5-HT by NMR [32,33]. The structure of compound 7 was elucidated by NMR data as detailed below: ^1^H NMR (400 MHz, D_2_O) *δ* 7.41 (1H, d, *J* = 8.8 Hz, H-7), 7.27 (1H, s, H-2), 7.09 (1H, d, *J* = 2.0 Hz, H-4), 6.87 (1H, dd, *J* = 8.8, 2.0 Hz, H-6), 3.30 (2H, t, *J* = 7.2 Hz, H-α), 3.10 (2H, t, *J* = 7.2 Hz, H-β). ^13^C NMR (125 MHz, D_2_O) *δ* 148.3 (C-5), 131.2 (C-7a), 126.6 (C-3a), 124.9 (C-2), 112.4 (C-7), 111.4 (C-6), 108.0 (C-3), 102.0 (C-4), 39.2 (C-α), 22.1 9 (C-β) (Supplementary Materials Figure S3).

### 2.3. Antimicrobial Activity

VBV displayed a significant effect against gram-positive (*Staphylococcus aureus [S. aureus]*) and gram-negative (*Escherichia coli [E. coli]*) bacterial strains. Of note, VBV showed strong antibacterial activity against *Propionibacterium acnes* (*Pr. acnes*) and no activity against *Pseudomonas aeruginosa* (*P. aeruginosa*). VBV markedly inhibited *Pr. acnes* growth with inhibition zones of 19.0 ± 0.3 mm, minimum inhibitory concentration (MIC) of 12.5 µg/mL, and minimum bactericidal concentration (MBC) of 12.5 µg/mL. The corresponding inhibition zones, MICs, and MBCs are listed in Table 1.

### 2.4. Antioxidant Activity

**Figure 3:**
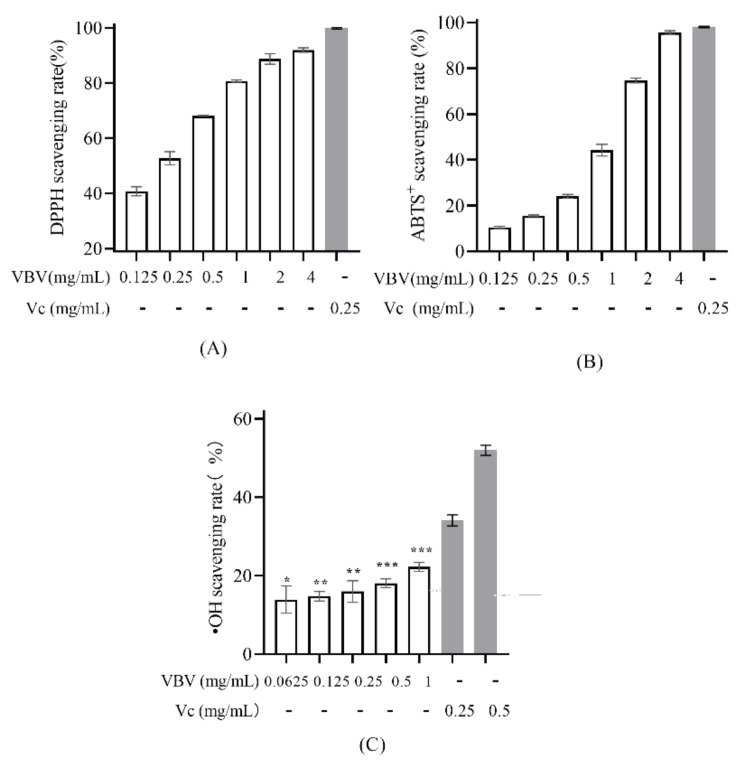
DPPH radical scavenging activity (**A**), ABTS radical scavenging activity (**B**), and •OH radical scavenging activity (**C**) were analyzed to evaluate the antioxidant properties of VBV. Each experiment represents the mean values of three independent experiments. * *p* < 0.05, ** *p* < 0.01, *** *p* < 0.001 compared with negative controls.

The VBV exhibited strong antioxidant abilities against DPPH radicals and ABTS^+^ radicals and showed concentration-dependent scavenging activity against DPPH and ABTS+ radicals with half-maximal inhibitory concentration (IC50) values of 0.178 and 1.41 mg/mL, respectively (Figure 3A,B). In addition, although a low concentration of VBV (0.0625 mg/mL) had a significant scavenging effect on •OH radical, VBV showed a weak concentration dependence (Figure 3C).

### 2.5. Anti-Inflammatory Assay

**Figure 4:**
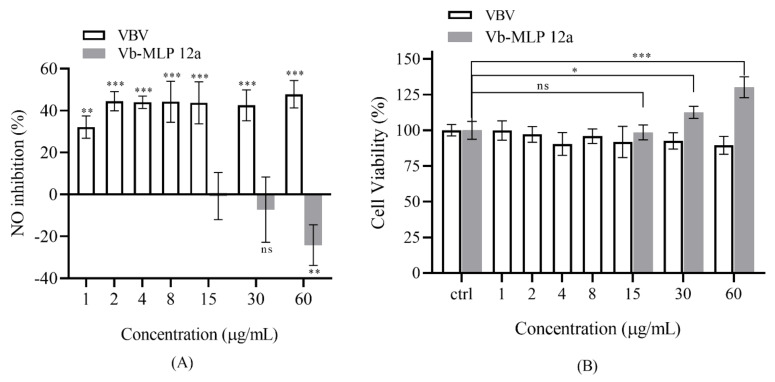
VBV and Vb-MLP 12a suppressed LPS-mediated NO release without any cytotoxicity. VBV had potent anti-inflammatory activity, and Vb-MLP 12a showed pro-inflammatory activity (**A**). VBV did not show any cytotoxic effect, and Vb-MLP 12a could promote RAW 264.7 cell proliferation (**B**). Data are expressed as mean ± SD. Each experiment represents the mean values of six independent experiments. * *p* < 0.05, ** *p* < 0 01, *** *p* < 0 001 compared with negative control.

In our study, we found that VBV potently suppressed LPS-induced inflammation (Figure 4A) without showing any cytotoxic effect at the indicated concentrations (Figure 4B). Notably, VBV showed strong anti-inflammatory activity even at a low concentration (1 µg/mL) and had a weak concentration-dependent effect (Figure 4A). Interestingly, Vb-MLP 12a, a major peptide from VBV, promoted the release of NO at high concentrations (Figure 4A) (30 µg/mL) because it can promote RAW 264.7 cell proliferation (Figure 4B).

### 2.6. Cytotoxic Activity Assay on Cancer Cells

**Figure 5:**
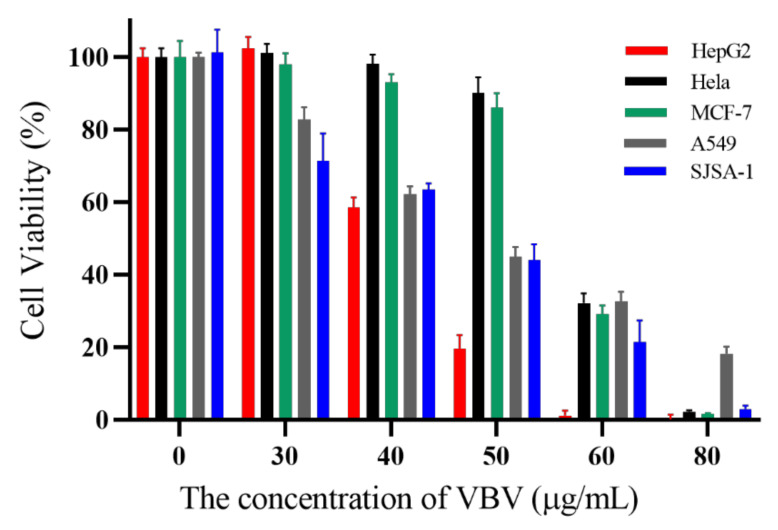
Cytotoxic activity of VBV on different human cancer cells. The IC50 values of VBV on HepG2, Hela, MCF-7, A549, and SJSA-1 cancer cells were 42, 58, 54, 48, and 46 µg/mL, respectively. Data are expressed as mean ± SD. Each experiment represents the mean values of six independent experiments.

VBV showed different degrees of inhibitory activity against five tumor cell lines (HepG2, Hela, MCF-7, A549, and SJSA-1). The IC50 value of VBV on different cancer cells ranged from 40 µg/mL to 60 µg/mL (42, 58, 54, 48, and 46 µg/mL, respectively) (Figure 5). The viability of HepG2 cells was most affected by the change in VBV concentration.

## 3. Discussion

We characterized the compositions and biological properties of VBV. Similar to most other wasp species, hyaluronidase A, phospholipase A1 (two isoforms), and allergen 5 protein are present in VBV [7,14], suggesting that it has similar allergens with other species. This finding also implies that VBV has similar toxicity, such as causing hemolysis and hypersensitivity.

Of note, two new proteins (protein 5 [16.68 kDa], protein 6 [11.35 kDa]) were found in VBV, which could not be identified through the NCBI database. It is worth mentioning that although they can match some proteins in the *Apocrita* database, the number of matched peptides is insufficient, and the results are unreliable. In addition, although the structure and activity of these two proteins were not characterized individually in this study, they were considered characteristic proteins of VBV or might even have a specific activity. Moreover, they can be applied in the identification of VBV at the present stage. With the in-depth development of VBV, researchers should pay more attention to these two proteins.

In addition, compared with the reported literature [32,33], we identified components 7 and 8 as 5-HT and Vb-MLP 12a, respectively. Vb-MLP 12a is a mastoparan-like peptide first reported in *Vespa* magnifica venom [31]. A high amount of active components (Vb-MLP 12a and 5-HT) in VBV, implying that it has clear development potential and application value because the mastoparan family and 5-HT have been reported to possess a broad spectrum of biological activities, such as antimicrobial, anticancer, and antioxidant [5,20,21,22,23]. Furthermore, we carried out semi-preparative HPLC to purify and obtain high-purity Vb-MLP 12a and 5-HT. This method provides a new way to obtain high-purity Vb-MLP 12a and 5-HT and promotes their development and application.

VBV was then tested for different biological activities, trying to demonstrate its potential application value as a whole. According to previous reports on mastoparan-like peptide 12a of *V. magnifica* venom [31], we speculated that VBV has antimicrobial effect against gram-positive (*S. aureus*) and gram-negative bacterial strains (*E. coli*) because it contains Vb-MLP 12a, a mastoparan-like peptide 12a from VBV. Chen et al. [29] found mastoparan (MP-VB1) and chemotactic peptide (VESP-VB1) in VBV from northern China, which have a significant antimicrobial effect against *P. aeruginosa* (MIC 120 and 3.75 µg/mL, respectively). However, in our study of VBV from South China, we observed no inhibitory activity against *P. aeruginosa* even at VBV concentrations up to 10 mg/mL. Combined with the results of component analysis, one possibility is that our VBV might not have MP-BV1 and VESP-VB1 due to regional divergence. To our knowledge, this study is the first to investigate and find that *Vespa* venom has an extremely significant antimicrobial effect against anaerobic bacteria such as *Pr. acnes*. It provides a new idea for the application of *Vespa* venom and the treatment of acne.

As for the antioxidant effect of VBV, we found that *V. bicolor* crude venom showed strong antioxidant activity. We speculate that it might be due to the presence of mastoparan and 5-HT, which has already been identified as the major active compound with free radical scavenging activity in the venom of *V. velutina nigrithorax* [5,24].

We also found that VBV had significant anti-inflammatory activity against LPS-induced RAW 264.7 cells at low concentrations (1 µg/mL), but this effect was not related to the individual components identified in VBV. On the contrary, a previous study has shown that the phospholipase A1 of *Vespa* venom could hydrolyze the phospholipids and produce arachidonic acid with pro-inflammatory activity, especially oxygen free radicals, which can also activate membrane phospholipase to promote inflammation [34]. Combined with the analysis of VBV composition, we speculated that it might be due to the indirect inhibition of the pro-inflammatory activity of PLA1 by the antioxidant activity of 5-HT, or that some unreported components have anti-inflammatory activity, such as component 5 and 6. To our knowledge, this study is the first to show the anti-inflammatory activity of VBV, suggesting that it can be used in the treatment of rheumatism and is a potential resource for the development of anti-inflammatory drugs. Moreover, we first reported that Vb-MLP 12a, a major peptide of VBV, has significant effect to promote RAW 264.7 cell proliferation, implying that VBV has promising prospects in medical application, such as enhanced immune function.

Finally, in this preliminary study of the cytotoxic activity of VBV against different tumor cells, the tumor cytotoxic activity may be associated with Vb-MLP 12a, a mastoparan-like peptide. Studies have revealed that mastoparan has cytotoxic activity against tumor cells of leukemia, myeloma, breast cancer, and melanoma [35]. Moreover, mastoparan can insert into the membrane bilayer causing lysis or interact with G proteins on the cytoplasmic face, thus inducing cell death by necrosis and/or apoptosis [36,37]. In addition, mastoparan has selective cytotoxic activity due to its overall positive charge structure, promoting binding to the negatively charged cell membrane of tumor cells [38,39]. Therefore, Vb-MLP 12a may be one of the main active components in VBV that promote apoptosis or necrosis of tumor cells. In this study, VBV has cytotoxic activity against different tumor cells (HepG2, Hela, MCF-7, A549, SASJ-1), especially HepG2 (human liver cancer cells), suggesting that Vb-MLP 12a and/or even VBV is a potential resource for the treatment of liver cancer.

## 4. Conclusions

We characterized the compositions and biological characteristics of VBV for the first time. In terms of protein characterization, we demonstrated that VBV has similar allergens to other wasps, such as hyaluronidase A, phospholipase A1 (two isoforms), and allergen 5 protein. In addition, we discovered two unreported proteins (protein 5 [16.68 kDa], protein 6 [11.35 kDa]) in VBV, which are considered to be characteristic proteins of VBV or even have a specific activity, and can be used in the identification of VBV at the present stage. In terms of chemical characterization, we first reported that VBV contains a large number of Vb-MLP 12a and 5-HT, which can be obtained in high purity using the semi-preparative HPLC technique. In the biological activity test of VBV, we found that VBV had significant anti-anaerobic, antioxidant, anti-inflammatory, and cytotoxic activities. Furthermore, the Vb-MLP 12a of VBV promotes RAW 264.7 cell proliferation, thus showing pro-inflammatory activity. These findings suggest that VBV has a clear development potential and application value. Despite the identification of the major components in VBV, some other unreported compounds, which play a synergistic/antagonistic role, could be involved in the bioactivities of VBV. Future studies will focus on the unknown components that may have special biological properties and potential application value.

## 5. Materials and Methods

### 5.1. Venom

Different batches of VBV were collected from different provinces (Guangdong, Guangxi, Guizhou) of China from July to November in 2020. The *Vespa* species was identified by Professor Guo Yunjiao (Institute of Edible Insects, Dehong Normal University, Mangshi, Dehong Prefecture Yunnan, China) as *Vespa bicolor* Fabricius. The crude venom was obtained by electrical stimulation, dissolved in ultrapure water, and centrifuged at 3000 rpm for 3 min. Subsequently, the supernatant was freeze-dried and kept at −80 °C.

### 5.2. Protein Characterization of VBV

#### 5.2.1. Protein Analysis of VBV by SDS-PAGE

The proteins of VBV were separated by SDS-PAGE [40,41]. The samples were solubilized in ultrapure water, heated at 100 °C for 3 min in a water bath, and loaded on SDS-PAGE gel (15% *w*/*v*). The electrophoresis was set for 30 min with 80 V, followed by 150 V for 1 h. Then, the samples were stained with Coomassie Blue. In addition, 1 µL of 1 mg/mL VBV aqueous solution was placed on the target plate, dried at room temperature, and add with 1 µL of SA matrix (sinapic acid). Then the sample was dried at room temperature, and analyzed by MALDI-TOF-MS (Omni Flex MALDI-TOF MS, VSL-337i, Bruker, Billerica, MA, USA).

#### 5.2.2. Identification of Proteins of VBV by LC–MS/MS

In brief, the VBV solution or protein bands were digested by trypsin (trypsin to protein mass ratio is 1:50) overnight at 37 °C. After purification and drying, the samples were dissolved with a solution (2% acetonitrile and 0.1% formic acid) and analyzed by LC–MS/MS, which was equipped with an Acclaim PepMap RSLC C18 (300 µm id × 5 mm, 5 µm, 100 Å, Thermo, 160454) and Acclaim PepMap 75 µm × 150 mm C18 (3 µm, 100 Å Thermo, 160321) analytical reversed-phase column [12,42]. The flow rate of this separation was 350 nL/min. Mobile phase A (0.1% formic acid/water) and mobile phase B (0.1% formic acid/80% acetonitrile) were used. The gradient program was as follows: 0–5 min, 5–5% B; 5–45 min, 5–50% B, 45–50 min, 50–90% B, 50–55 min, 90–90% B, 55–60 min, 90–5% B. The identification of the peptides was carried out using a Q Exactive mass spectrometer (Thermo Scientific, Waltham, MA, USA). The scan range of the mass spectrometer ranged from *m*/*z* 350 to 1800. MGF format files were obtained from raw mass spectrometry data by MM File Conversion software and retrieved through the NCBI database by MASCOT (http://www.matrixscience.com/ accessed on 15 July 2021).

### 5.3. Chemical Characterization of VBV

#### 5.3.1. Chemical Analysis of VBV by HPLC

VBV was analyzed by HPLC system (Shimadzu, Kyoto, Japan) including SPD-M20A UV-VIS detector, SIL-20A injection valve, and LC-20AT pump. The obtained data were analyzed at 30 °C on an Ultimate LP-C18 (4.6 mm× 250 mm, 5 µm, 300 A) column. The analysis parameters were as follows: the detection wavelength was 276 nm, the flow rate was 1 mL/min, the injection volume of samples was 20 µL (2 µg/µL), mobile phase A was (0.1% trifluoroacetic acid/water), and mobile phase B was (0.1% trifluoroacetic acid/acetonitrile) [33,43]. The gradient program was as follows: 0–45–60 min, 3–70–70% B. Additionally, the main components of VBV in the chromatograms were used in a semi-preparative column (LP-C18, 10.0 mm × 250 mm, 5 µm, 300 A).

#### 5.3.2. Identification of Chromatographic Peaks

The component sample solution of VBV was identified by MALDI-TOF-MS or NMR. In brief, 1 µL of 1 mg/mL sample aqueous solution was placed on the target plate, dried at room temperature, and added with 1 µL of CHCA matrix (α-cyano-4-hydroxycinnamic acid). Then, the sample was dried at room temperature and analyzed by MALDI-TOF-MS (Omni Flex MALDI-TOF MS, VSL-337i, Bruker, Billerica, MA, USA). In addition, 1H and 13C-NMR spectra were obtained in D2O by using an Avance III 400 MHz NMR spectrometer (Bruker BioSpin GmbH, Rheinstetten, Germany).

### 5.4. Antibacterial Activity Assay

Gram-positive bacterium *S. aureus* (ATCC25923), gram-negative bacterium *P. aeruginosa* (ATCC27853) and *E. coli* (ATCC25922), and anaerobe *Pr. acnes* (ATCC6919) were used for the antimicrobial assays. The aerobic bacteria (*S. aureus, P. aeruginosa, E. coli*) were resuspended in Müller–Hinton broth, incubated overnight at 37 °C, and stored at 4 °C. The anaerobe *Pr. acnes* was uniformly spread in a *Clostridium* growth medium, incubated at 37 °C with an anaerobic environment for 48–72 h, and stored at 4 °C.

#### 5.4.1. Inhibition Zone Diameter Measurements

The antibacterial activities of the VBV were analyzed by disc diffusion assay. The inoculum (200 µL) was distributed uniformly on the surfaces of each plate. A blank drug disc (6 mm) was placed on the surface of the plate, and 10 mg/mL of VBV solution was added. Physiological saline was used as a blank control. Bacteria were incubated with corresponding conditions for 24–48 h. The size of the clear zone that was formed on the surface of each plate reflects the antibacterial activity of VBV.

#### 5.4.2. Determination of MIC and MBC

The minimum inhibitory concentration (MIC) and the minimum bactericidal concentration (MBC) of VBV were determined using CLSI recommendations. The concentration of VBV in a 96-well plate (1200 µg/mL to 0.78 µg/mL) was diluted with broth and incubated with the bacteria (equivalent to 1.5 × 10^8^ CFU/mL) under corresponding conditions for 24–48 h [6,29,44]. MIC is the concentration at which the growth of bacteria is not visible to the naked eye. Then, 5 µL of the supernatant of a 96-well plate without bacterial growth was spread on a new culture. The plates were incubated under the corresponding conditions for 48 h [45,46]. MBC is the concentration at which the growth of bacteria is not visible to the naked eye.

### 5.5. Antioxidant Activity Assay

#### 5.5.1. 1,1-Diphenyl-2-picrylhydrazyl (DPPH) Assay

Different concentrations of VBV solution (0.125, 0.25, 0.5, 1, 2, and 4 mg/mL) were mixed with an equal volume of DPPH solution (400 µM) in a 96-well plate, incubated at 37 °C for 30 min, and then measured at 517 nm [5,47,48]. In addition, vitamin C (0.25 mg/mL), ethanol solutions, and DPPH solution were set as the positive controls, negative controls, and blank, respectively. The DPPH radical scavenging rate was calculated as follows: ((O.D. blank-O.D. sample)/O.D. blank) × 100%.

#### 5.5.2. 2,2′-Azino-bis(3-ethylbenzothiazoline-6-sulfonic Acid) Diammonium Salt (ABTS) Assay

ABTS assay was carried out using an antioxidant capacity assay kit following the manufacturer’s protocol (Beyotime, Shanghai, China). About 10 µL of the VBV solution at different concentrations (0.125, 0.25, 0.5, 1, 2, and 4 mg/mL) was mixed with 200 µL of ABTS^+^ solution on a 96-well plate, incubated at room temperature in the dark for 10 min, and then measured at 734 nm. Additionally, vitamin C (0.25 mg/mL), aqueous solution, and ABTS^+^ solution were set as the positive control, negative control, and blank, respectively [47,48]. The ABTS^+^ radical scavenging rate was calculated as follows: ((O.D. blank-O.D. sample)/O.D. blank) × 100%.

#### 5.5.3. •OH Radical Scavenging Ability

About of 52 µL of VBV at different concentrations (0.0625, 0.125, 0.25, 0.5, and 1 mg/mL), 8 µL of FeSO_4_ solution (18 mM), and 8 µL of salicylic acid–methanol solution (18 mM) were mixed in a 96-well plate. In addition, vitamin C (0.25 and 0.5 µg/mL) and aqueous solution were set as the positive control and negative control, respectively. The reaction system without FeSO_4_ solution served as blank. All plates were added 32 µL of 0.1% H_2_O_2_ solution to initiate the reaction, incubated for 30 min, and measured at 510 nm [47]. The •OH radical scavenging rate was calculated as follows: (1-(O.D. sample-O.D. blank-)/(O.D. negative-O.D. blank)) × 100.

### 5.6. Anti-Inflammatory Activity Assay

RAW 264.7 cells (ATCC, TIB-71) were cultured in DMEM combined with 10% FBS, 100 IU/mL penicillin, and 100 µg/mL streptomycin sulfate, which was incubated at 37 °C with a 5% CO_2_ condition. Different concentrations of the sample solution were tested in the inflammatory model mediated by LPS (1 µg/mL) that was incubated in a 96-well plate with 4 × 10^4^ cells/well, and incubated at 37 °C for 24 h. In addition, the group without VBV and the group without LPS and VBV were set as the negative control and blank, respectively. The anti-inflammatory activity was determined using the Griess reagent kit following the manufacturer’s protocol (Beyotime, Shanghai, China). Furthermore, a cytotoxicity study was assessed using the CCK8 kit following the manufacturer’s protocol.

### 5.7. Cytotoxic Activity Assay on Cancer Cells

HepG2 (human liver cancer cells, ATCC HB-8065), MCF-7 (human breast cancer cells, ATCC HTB-22), Hela (human cervical cancer cells, ATCC CCL-2), SJSA-1 (human osteosarcoma cells, ATCC CRL-2098), and A549 (human lung cancer cells, ATCC CCL-185) cancer cell lines were cultured in DMEM or RPMI-1640 combined with 10% FBS, 100 IU/mL penicillin, and 100 µg/mL streptomycin sulfate, which was incubated at 37 °C with a 5% CO_2_ condition. After culturing the tumor cells (5 × 10^3^ cells/plate) for 24 h, the cells were treated with VBV solution (100 µL) at concentrations of 30, 40, 50, 60, and 80 µg/mL. In addition, the group without VBV and the group without cells were set as the negative control and blank, respectively. At 24 h following treatment, cytotoxic activity was assessed using the CCK8 kit following the manufacturer’s protocol.

### 5.8. Statistical Analysis

The statistical significance of samples was analyzed using one-way analysis of variance, and post-hoc analyses were performed using Tukey’s HSD. The results are expressed as mean values ± standard deviation (SD). Statistical significance was considered at * *p* < 0.05, ** *p* < 0.01, and *** *p* < 0.001.

## 6. Patents

This study produced two patents: 1. A fingerprint detection method for *Vespa bicolor* venom, China, CN113109466A; 2. A method for rapid identification of *Vespa bicolor* venom authenticity and determination of adulteration amount, China, CN112730571A.

## Figures and Tables

**Table 1:** Inhibition effect of VBV on different strains of bacteria.

Microorganisms	Inhibition Zone ^1^ (mm)	MIC ^2^ (µg/mL)	MBC ^3^ (µg/mL)
*S. aureus*	12.3 ± 0.2	25	50
*E. coli*	10.2 ± 0.5	100	200
*P. aeruginosa*	ND ^4^	ND	ND
*Pr. acnes*	19.0 ± 0.3	12.5	12.5

^1^ Growth inhibition effects of VBV were determined by the disc diffusion method (10 mg/mL). The inhibition zone was shown to represent the mean ± SD. ^2,3^ The MIC and MBC were obtained using serial dilutions. ^4^ No detectable antimicrobial activity. These concentrations represent the mean values of three independent experiments.

## Data Availability

Processed data and analysis scripts are available on https://www.iprox.cn/ ID: IPX0003968000.

## Supplementary Materials

The following supporting information can be downloaded at https://www.mdpi.com/article/10.3390/toxins14010059/s1. Figure S1. Four proteins in VBV were identified by LC–MS/MS. Figure S2. Proteins 5 and 6 match results in the *Apocrita* database. Figure S3. Component 7 of VBV was identified as 5-hydroxytryptamine (5-HT) by NMR. Figure S4. Component 8 of VBV was identified as Vb-MLP 12a by LC–MS/MS.
