# Supplementary material for: Characterization of the Composition and Biological Activity of the Venom from Vespa bicolor Fabricius, a Wasp from South China
Source: Toxins (Basel). 2022 Jan 14;14(1):59. doi: 10.3390/toxins14010059 (PMC8777732; doi:10.3390/toxins14010059)
Supplement: Supplementary file 1 [file toxins-14-00059-s001.zip › toxins-1536624-supplementary.pdf]

toxins

MDPI

Supplementary Materials: Characterization of the Composition and Biological Activity of the Venom from *Vespa bicolor* Fabricius, a Wasp from South China

Yonghua Wu, Yu Zhang, Danqiao Fang, Jing Chen, Jingan Wang, Lin Jiang and Zhufen Lv

A. Hyaluronidase A

1 NLNR**TNWPK**K IFNIYWNVPT YFCHQHDVYF NELTK**FDIKY** **NPK**GNYR**GDT**
51 **ISLFYDPGNF** **PAMVPLK**NGT YDIRNEGVPQ KGNITVHLQQ FTK**ELDEIYP**
101 **KKISGGIGVI** **NFNKWRPIFR** RNVNNLK**INK** **EVSIDLVRK**E HPK**WDKSMIE**
151 **TEASNRFEK**S AR**IFMEKTLK** LAKDIRNKNK WGYHGYPYCP TASTGNPSFD
201 CDALAMNEND K**LSWLFKYQD** **VLLPSVYVK**H VLKPEEKIGL VRGSVKEAVR
251 ISKKFEHLPK VLSYWWYAYE DK**MDTFLTET** **DVKNTFR**EIL INGGDGIIIW
301 GTMHDLNKEK CEKLK**QYLST** **ILGPIAFK**VM EAVKK**RTPLN** **F**

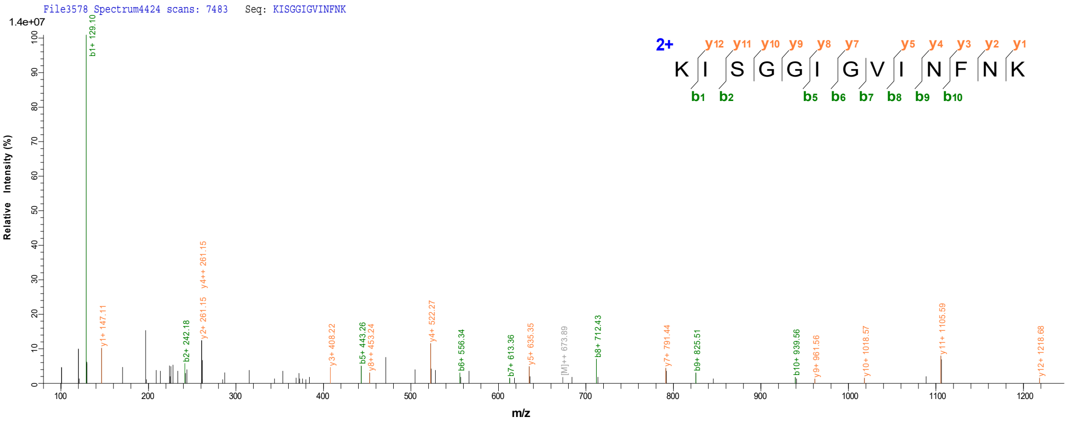

B. Allergen 5

1 NNYCKIKCRS GIHTLCKYGT STKPNCGRSV VKASGLTKAE KLEILKQHNE
51 FRQKVARGLE TRGNPGPQPP AKSMNTLVWN DELAQIAQVW ASQCKYGHDN
101 CRNTAKYLVG QNIAEQSTTA ASFEPVSNMV KMWSDEVKDY QYGSSKNKLN
151 DVGHYTQMVW AKTKEIGCGN IKYIENGWHH HYLVCNYGPA GNIGNEPIYE
201 KK

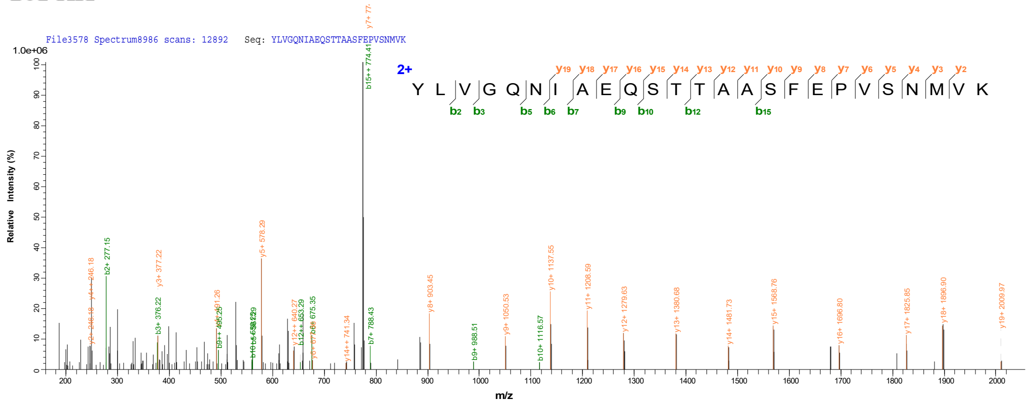

C. Phospholipase A1

1 GLLPKCKLVP EQISFILSTR ENRNGVFLTL DSLKKGGILN KSDLSSTQVV
51 FLIHGFISSA NNSNYMDMTK ALLEKNDCMV ISIDWRNGAC TNEFQILKFI
101 GYPKAVENTR TVGKYIADFS KLLMQKYKVS LANIRLIGHS LGAQIAGFAG
151 KEYQKFKLGK YPEIIGLDPA GPLFKSNDCS QRICETDAHY VQIIHTSNNL
201 GTERTLGTVD FYMNNGYNQP GCYYSFIGET CSHTRAVQYF TECIRHECCL
251 IGVPQSKNPQ PVSKCTRNEC VCVGLNAKRY PKTGSFYVPV ESKAPYCNNK
301 GKKI

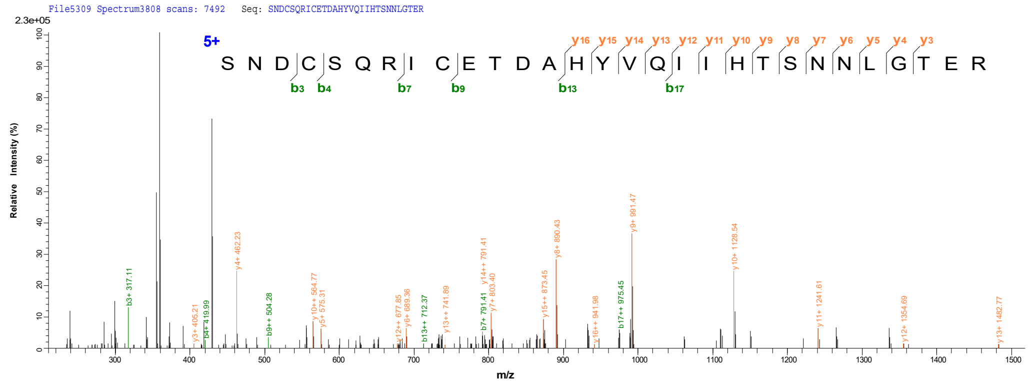

D. Phospholipase A1

1 **FNPCPYSDDT** **VKMIILTR**EN K**KHDFYTLDT** **IK**K**HNEFK**KS TIKHQVVFIT
51 HGFTSSADTE NFLAMAKALS DKGNYLVILI DWRVAACTEE MSGIQLAYYS
101 YAASNTR**LVG** **NYIATVTKML** **VQKYNVPMAN** **IRLIGHSLGA** **HTSGFAGKKV**
151 **QELGLGKYSE** **IIGLDPAGPS** **FK**SNDCSERI CKTDAHYVQI IHTSNHLGTL
201 VTLGTVDFMN NGYNQPGCGL PLIGETCSHT RAVK**YFTECI** **KHECCLIGVP**
251 **QSK**KPQPVSK **CTRNECVCVG** **LNAKTYPKTG** **SFYVPVESKA** **PYCNNK**GKII

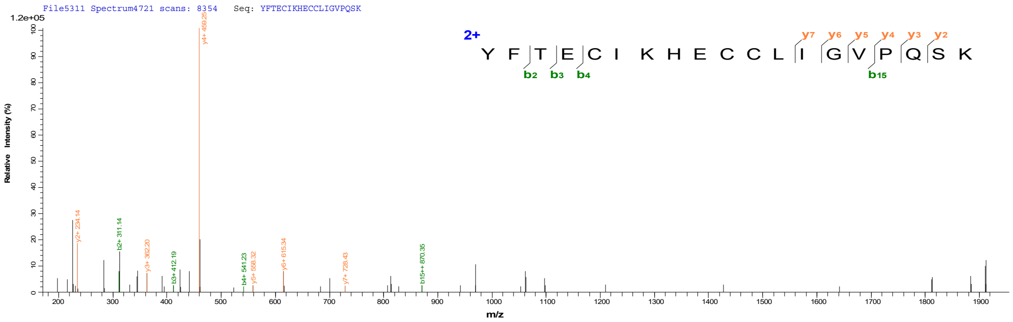

Figure S1. Four proteins in VBV were identified by LC-MS/MS (**A–D**), and the respective secondary mass spectrograms of proteins 1 to 4 are displayed below. **A**) Complete coding sequence of hyaluronidase A is shown, and a sequence coverage of the matched peptides of protein 1 in hyaluronidase A is highlighted. **B**) Complete coding sequence of allergen 5 is shown, and a sequence coverage of the matched peptides of protein 4 in allergen 5 is highlighted. **C**) Complete coding sequence of phospholipase A1 is shown, and a sequence coverage of the matched peptides of protein 2 in phospholipase A1 is highlighted. **D**) Complete coding sequence of phospholipase A1 protein is shown, and a sequence coverage of the matched peptides of protein 3 in phospholipase A1 is highlighted.

A. Peptidyl-prolyl cis-trans isomerase

1 MKLLLFLGLV AAASCANNAN GPKVTDKVWF DIKIGDADAG RIEIGLFGKT
51 VPKTVKNFVE LAKKPEGEGY KGSVFHRVIK DFMIQGGDFT KGDGTGGRSI
101 YGDRFADENF KLKHYGAGWL SMANAGKDTN GSQFFITVKQ TPWLDGRHVV
151 FGKIIKGMDI VRKIEKTNTD SRDRPQEDVV IADSGAETVA DPFSVSKDDA
201 IN

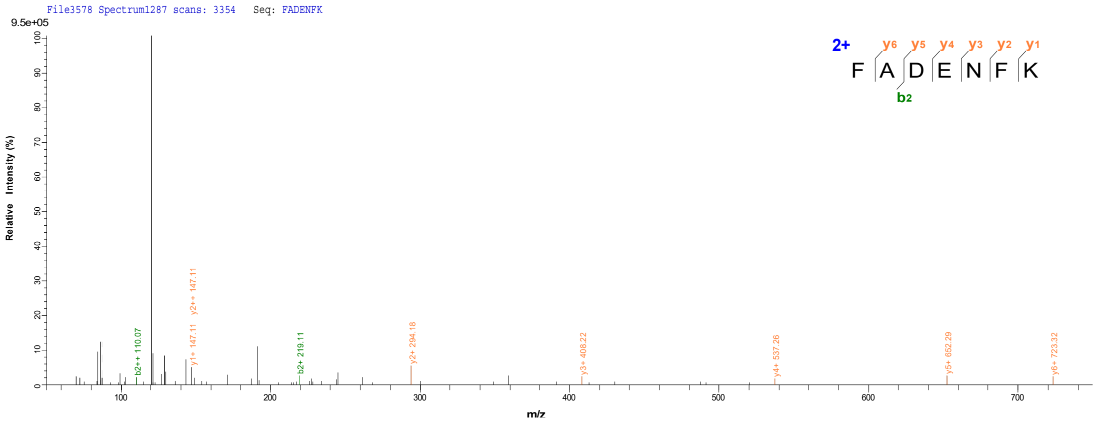

B. Uncharacterized protein

1 MSYDLRSGMW YFDGGQPEVN LRFTRQYRLL LAPPRALPKV PRGVPSXXXX
5 1 XXMVARALII LIINRLEQLT PAPYTKGITD LRGGSRQNRS ANNRLPLIAK
101 QSLLRNVVIT PL

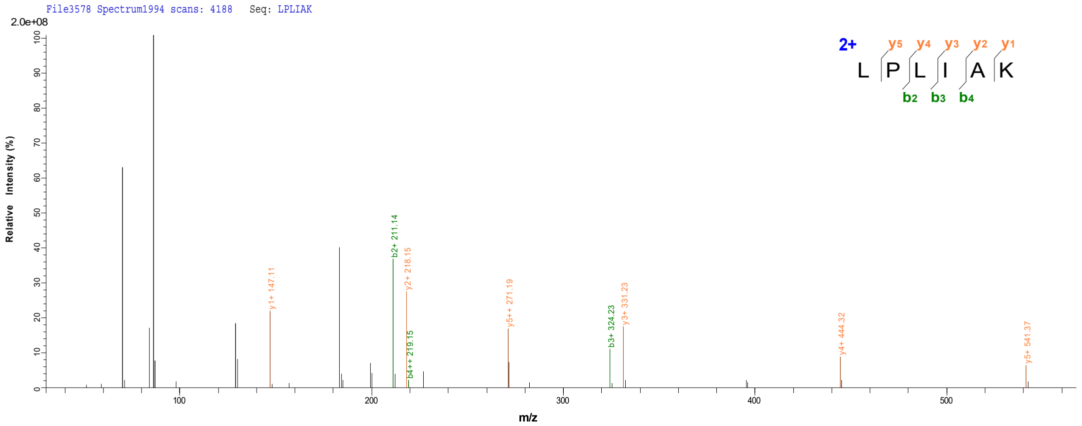

Figure S2. Proteins 5 and 6 match results in the *Apocrita* database. **A**) Complete coding sequence of peptidyl-prolyl cis-trans isomerase is shown, and a sequence coverage of the matched peptides of protein 5 in peptidyl-prolyl cis-trans isomerase is highlighted. **B**) Complete coding sequence of uncharacterized protein is shown, and a sequence coverage of the matched peptides of protein 6 in uncharacterized protein is highlighted.

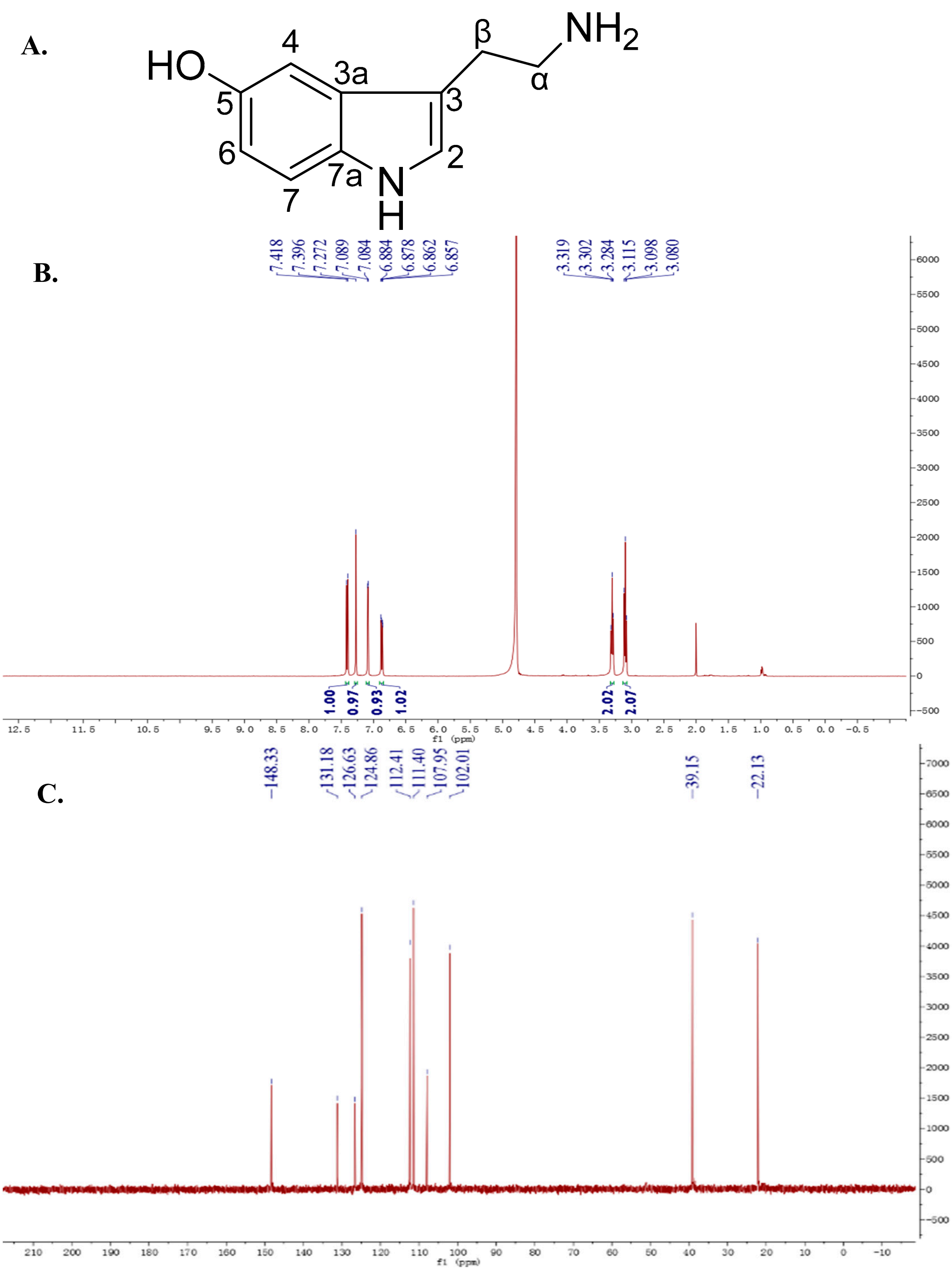

Figure S3. Component 7 of VBV was identified as 5-hydroxytryptamine (5-HT) by NMR. **A**) Structural formula of 5-HT. **B**) ^{1}H NMR spectrum of 5-HT in D_2O (400 MHz). **C**) ^{13}C NMR spectrum of 5-HT in D_2O (500 MHz).

Vb-MLP 12a

1 INWKGIAAMA KKLL

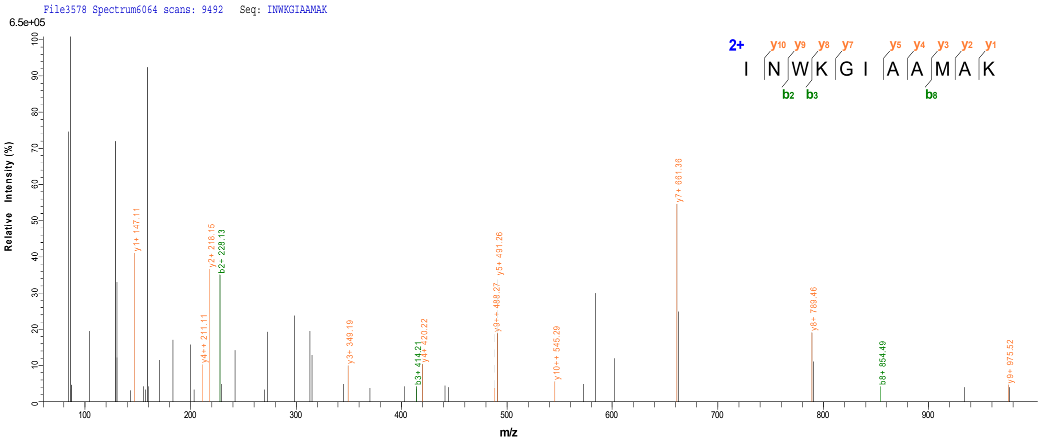

Figure S4. Component 8 of VBV was identified as Vb-MLP 12a by LC-MS/MS. The sequence coverage of the matched peptides is highlighted, and the secondary mass spectrograms of Vb-MLP 12a are displayed below.
